# Supplementary material for: A UG5 reverse transcriptase-nitrilase antiviral module confers phage immunity in the plant symbiont Sinorhizobium meliloti
Source: Microbiol Spectr. 2026 Jul 10;14(8):e00381-26. doi: 10.1128/spectrum.00381-26 (PMC13436386; doi:10.1128/spectrum.00381-26)
Supplement: Supplementary file 1 [file spectrum.00381-26-s0002.docx]

**Supplementary material**

**Supplementary Figures**

**Figure S1. Metagenomic distribution of UG5-associated nitrilase domains across environmental biomes.** UG5-associated nitrilase-domain sequences were identified by HMM-based searches against the MGnify protein database using a profile derived from the UG5 nitrilase domain. An initial set of 2,497 hits was retrieved and subsequently filtered using a stringent significance threshold (E-value ≤ 1 × 10⁻⁵⁰), yielding 1,004 high-confidence sequences. These filtered hits were then mapped to sample-level biome annotations. The figure shows the global distribution of UG5-associated nitrilase-domain sequences across annotated biomes, together with a highlighted hybrid donut representation in which soil- and rhizosphere-derived metagenomes are shown explicitly and all remaining biomes are grouped as “Other.”

**Figure S2**. **US-align structural superposition of Structure_1 (red) onto Structure_2 (orange).** Structure_1 corresponds to chain A of RMO17-DRT11 (1005 residues), and Structure_2 to chain A of *Pseudomonas canadensis* (WP_14805088.1) belonging to nitrilase CL249 (307 residues). US-align produced an aligned region of 290 residues with an RMSD of 1.98 Å and a sequence identity of 0.262 across aligned positions. TM-score was 0.28047 when normalized by the length of Structure_1 (reference; L=1005, d0=10.56) and 0.88721 when normalized by the length of Structure_2 (L=307, d0=6.43).

**Supplementary Tables**

**Table S1**

**Table S2**

**Supplementary data**

**Supplementary Data 1**. UFBoot consensus tree (.contree; Newick) obtained with IQ-TREE2 for the nitrilase-domain phylogeny in Fig. 1A.
